# Resilience as a Determinant of Healthspan and Lifespan in Mice

**DOI:** 10.1093/geroni/igab046.621

**Published:** 2021-12-17

**Authors:** Nathan LeBrasseur

**Affiliations:** Mayo Clinic, Rochester, Minnesota, United States

## Abstract

Dynamic measures of physical resilience—the ability to resist and recover from a challenge—may be informative of biological age far prior to overt manifestations such as age-related diseases and geriatric syndromes (i.e., frailty). If true, physical resilience at younger or middle ages may be predictive of future healthspan and lifespan, and provide a unique paradigm in which interventions targeting the fundamental biology of aging can be tested. This seminar will discuss research on the development of clinically relevant measures of physical resilience in mice, including anesthesia, surgery, and cytotoxic drugs. It will further highlight how these measures compare between young, middle-aged, and older mice, and how mid-life resilience relates to later-life healthspan and even lifespan. Finally, it will provide insight into whether interventions targeting the biology of aging can modify physical resilience in mice.

